# Sinus Savvy: Exploring the Current Techniques of Maxillary Sinus Augmentation

**DOI:** 10.7759/cureus.61933

**Published:** 2024-06-08

**Authors:** Archita Chittoria, Yogendra Malviya, Aparajita Adurti

**Affiliations:** 1 Oral and Maxillofacial Surgery, Jaipur Dental College, Jaipur, IND; 2 Oral and Maxillofacial Surgery, School of Dental Sciences, Sharda University, Noida, IND

**Keywords:** maxillary sinus lift, sinus floor elevation, dental implants, sinus floor augmentation, bone morphogenetic protein (bmp)

## Abstract

Sinus ridge augmentation is a surgical procedure aimed at increasing the volume of bone in the posterior maxilla to permit successful dental implant placement. The current review article presents an overview of various techniques used for sinus ridge augmentation, including the lateral window technique, crestal approach, transalveolar technique, and piezoelectric osteotomy. The article examines the advantages and limitations of each technique, such as invasiveness, surgical difficulty, and the requirement for additional procedures. Additionally, the article discusses the factors that influence the success of the procedure, including patient age, residual bone height, and the kind of bone graft substance used. The review also emphasizes the importance of proper case selection, surgical planning, and postoperative care to ensure optimal outcomes. Overall, the article provides valuable insights into the current techniques used for sinus ridge augmentation, highlighting the need for further research to improve patient outcomes and the success of placing dental implants over the long run.

## Introduction and background

The maxillary sinuses are air-filled cavities located behind the cheeks and above the upper teeth. When teeth are lost, especially in the posterior maxilla, the bone that once supported them tends to shrink over time, leaving inadequate bone for stable implant anchorage. This limitation can compromise the success of dental implant treatment in these areas. Maxillary sinus augmentation addresses this issue by effectively increasing the bone volume and density in the posterior maxilla, creating a more favorable environment for the placement of dental implants. By elevating the sinus membrane and adding bone graft material beneath it, this procedure augments the bone height and width in the area, providing a stable foundation for dental implant placement.

The augmentation graft of the maxillary sinus was a crucial component of implant-directed maxillary reconstruction for over three decades [1]. Maxillary sinus augmentation, also known as sinus lift surgery, has evolved significantly over the years, from its early inception as a bone augmentation procedure pioneered by Tatum in the 1970s to the refined and sophisticated techniques utilized today. This procedure plays a pivotal role in creating a stable foundation for dental implants in the posterior maxilla, where bone quality and quantity are often compromised.

This article serves as a comprehensive exploration of the various maxillary sinus augmentation techniques currently employed in clinical practice. By delving into the intricacies of these techniques, we aim to provide a nuanced understanding of their principles, indications, advantages, and potential complications. Furthermore, we will highlight recent advancements and emerging trends that are reshaping the landscape of maxillary sinus augmentation, paving the way for improved outcomes and patient satisfaction. Through this exploration, we will elucidate the rationale behind each technique, ranging from lateral window and crestal approaches to innovative minimally invasive methods such as hydraulic sinus condensing and balloon sinus elevation.

The goal of the present article is to give an outline of the fundamental principles of maxillary sinus reconstruction, comprising the physiology and anatomy of the sinus, preoperative assessment, surgical signs, surgical procedures, and management strategies for issues.

## Review

Anatomy and physiology

**Figure 1:**
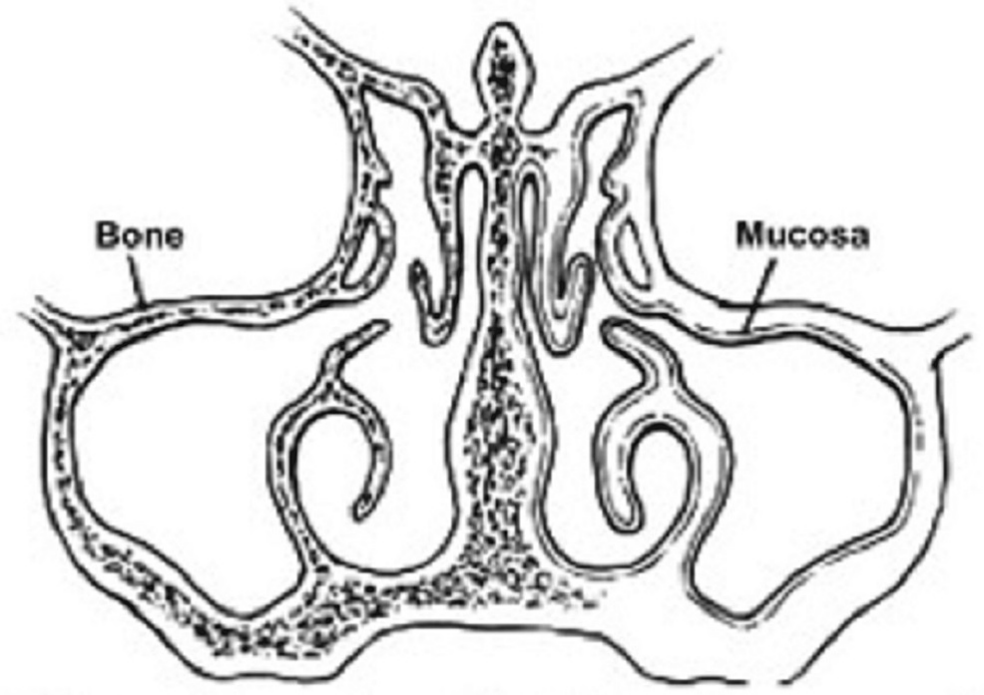
Coronal view of the ostiomeatal complex. The uncinate process lies in a sagittal plane. The maxillary sinus ostium drains into the infundibulum. Source: Stern and Green (2012) [2]; reproduced with permission from Elsevier

The maxillary sinuses are a pair of air-filled cavities located in the bilateral maxillae that are situated laterally to the nasal cavity, inferior to orbital floors, superior to maxillary teeth, and anterior to the infratemporal fossa (as shown in Figure 1).

The average volume of these sinuses is 12.5 mL, making them the biggest of the paranasal sinuses [3]. The maxillary sinuses are coated by the Schneiderian membrane, which is composed of a cell cambium layer (osteogenic periosteal layer) on the bone sides and ciliated respiratory epithelium (pseudostratified columnar epithelium) on the lumen side. The infraorbital nerve travels through the middle of the maxillary roof in a posterior-anterior direction. The floor of the canal is usually made up of thick bone, although, in a few instances, it may be absent, leaving just a thin mucosal layer between the sinus cavity and nerve [4]. The sinus ostium is situated on the medial wall of the nose above the uncinate procedure, and it communicates with the ethmoid infundibulum in the middle meatus on the walls of the nose. Bony, thin septae that span from the lateral to medial sinus wall might be found in up to 37% of people, with 45.9% in the middle, 22.5% in the third anterior, and 31.5% in the posterior sinus. One or two septae are found in 89% of individuals with septae [5].

The position as well as the presence of septa in the maxillary sinuses may have an impact on the treatment plan, and if they are not identified before the surgery, it can cause perioperative complications. This will be reviewed in detail later. The cilia of the Schneiderian membrane play a vital function in guiding the discharge of mucus and debris toward the ostium, thereby maintaining constant drainage in normally functioning sinuses. Certain medical conditions may increase the risk of chronic sinusitis in some patients. Allergic rhinitis can cause inflammation of the mucosa close to the ostium, resulting in inflammation and obstruction of mucous discharge, causing painful sinus pressure and stagnant fluid infection. Dysfunctional sinus cilia can cause a buildup of mucus and debris, which can cause infection since the sinuses are unable to remove the debris and regular discharge [6].

Indications and contraindications of sinus augmentation

The main reason for performing a sinus graft surgical procedure is to prepare for posterior maxilla implant reconstruction that has suffered from bone loss after tooth extraction and sinus pneumatization. In such cases, the bone has become too atrophic to support implants (as shown in Table 1). Sinus graft surgery is appropriate for a range of cases, including single-tooth as well as multi-tooth reconstruction and complete reconstruction of the posterior maxilla in edentulous patients.

**Table 1:** Indications for sinus lift surgery Source: Stern and Green (2012) [2]; reproduced with permission from Elsevier

Condition	Treatment
Edentulous maxilla with severely atrophic maxilla and pneumatized sinus	Open sinus lift via lateral maxilla sinus antrostomy; delayed implant placement
Edentulous maxilla with some remaining alveolar bone (0-4 mm)	Open sinus lift via lateral maxilla sinus antrostomy; delayed implant placement
Edentulous maxilla with some remaining alveolar bone (5-10 mm)	Open sinus lift via lateral maxilla sinus antrostomy; immediate implant placement
Single-tooth edentulous space with 5-7 mm of alveolar bone remaining	Open sinus lift via lateral maxilla sinus antrostomy; immediate implant placement
Single-tooth edentulous space with >8 mm of bone remaining	Open sinus lift via lateral maxilla sinus antrostomy or closed (crestal approach) osteotome technique; immediate implant placement

Preoperative assessment

A detailed dental, as well as medical history and physical examination, must be conducted prior to initiating maxillary sinus augmentation surgery. It is important to note any relevant positive history, like the latest upper respiratory disease, chronic sinus as well as sinusitis, otitis media, facial pain, past sinus or nasal surgery, prior maxillary reconstruction efforts, and smoking history. Studies have revealed that the complication risk for smokers who undergo sinus lift grafts is comparable to that of the common population, although there is an indication that smokers who have implants embedded in sinus transplanted bone have a greater rate of failure in comparison to non-smokers [7,8]. A preoperative CT scan is advised to evaluate the volume of bone already present, exclude preexisting sinus illness, and detect any bony septae [9].

Informed consent

Before beginning the maxillary sinus grafting surgery, it is crucial to have an informed consent discussion with the patient. This discussion should cover the benefits, risks, alternatives to the process, and the risks of alternatives. Common risks related to the procedure comprise bleeding, pain, swelling, graft failure, infection, and sensory changes to the second branch of the cranial nerve (V). It is important to note that smokers have a greater risk of implant failure, despite the potential success of the graft method. The eventual restoration of the edentulous maxilla is the main advantage of the procedure. The treatment can be substituted with a shorter implant, a three-unit bridge, zygomaticus implants, partial dentures, or angled implants. The risks of the alternatives should also be discussed. The patient must understand that the surgery is elective and that the decision to proceed is solely theirs after considering all the possible options. The expected timeframe for dental restoration after the procedure may exceed a year, and additional costs may be incurred. To help the patient understand the informed consent process, multimedia resources such as patient education videos, models, and radiographs can be used.

Surgical techniques

According to recent studies, maxillary sinus augmentation may be achieved using two techniques: the sinus intrusion osteotomy technique and the lateral window technique. These approaches for vertical augmentation in the oral cavity are regarded as the most reliable ones. These procedures can be carried out using many bone graft substances, comprising autogenous bone, xenograft, alloplastic, and allograft substances. Autogenous bone is thought to be an ideal graft for the sinus lift procedure because it offers enough feasible bone to initially support the implant and promote osseointegration [10]. Recent research has revealed that autogenous bone grafts are the better way because they maintain a sufficient alveolar ridge height for five to 10 years after initial insertion [11]. To enhance the amount of bone inserted into the maxillary sinus, demineralized freeze-dried bone may be combined with autogenous bone. Studies have revealed that adding demineralized freeze-dried bone to autogenous bone marginally reduces the level of bone attained; nevertheless, this change is insignificant clinically because the implants are still enclosed by bone [10].

Autogenous bone grafts are reflected to be unique as they contain endosteal osteoblasts, which give them the capacity to form bone directly. Along with osteoblasts, a corticocancellous graft also releases growth hormones and bone morphogenic proteins (BMPs), which promote the production of new bone [12]. The proximal tibia, calvarium, anterior iliac crest, and maxillofacial areas are a few anatomical locations that can be used to harvest bone transplants. These methods, however, are outside the purview of this essay, so we will not go into more detail about how they work.

**Figure 2:**
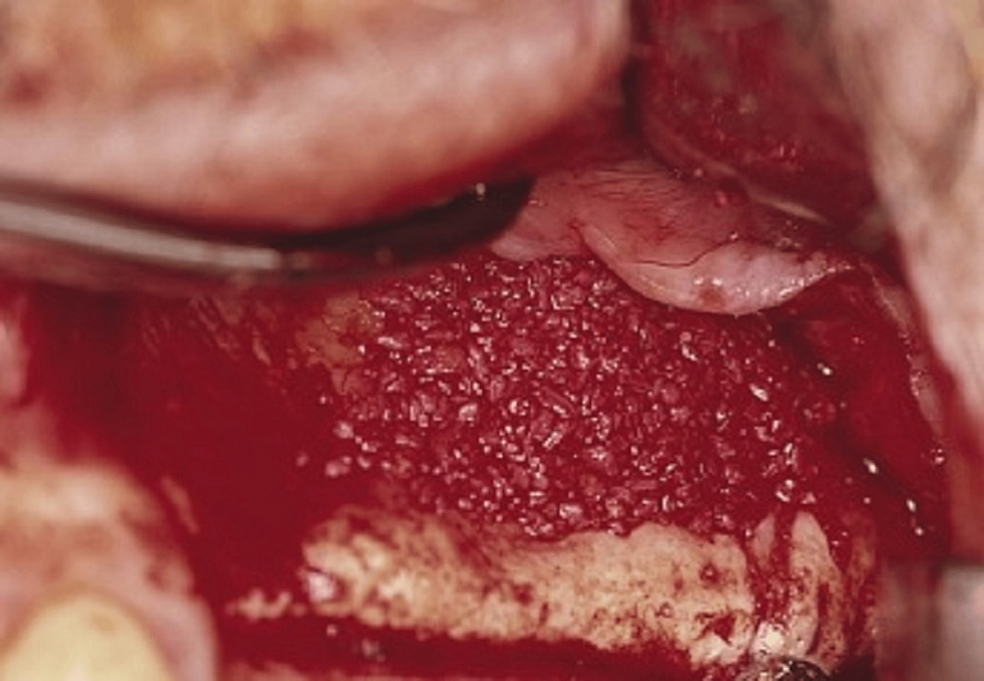
A bone graft composite is packed into the sinus site. After approximately six months, implants are placed, followed by a final restoration after another six months. Source: Stern and Green (2012) [2]; reproduced with permission from Elsevier

Several sites, including the maxillary tuberosity, ramus, symphysis, mandibular third molar site, and posterior maxilla, can be harvested for intraoral bone. Even though maxillary tuberosity provides a smaller amount of bone (1-2 mL), it is located in a similar surgical field as the lateral technique for the maxillary sinus and should be considered [13]. In the posterior maxilla, toward the hamular notch, a crestal incision is made to obtain the graft, along with any necessary vertical releasing incisions. The incision is prolonged posteriorly to reach the tuberosity when the maxillary sinus is to be accessed via the lateral window technique. The posterior maxilla is shown by raising a full-thickness mucoperiosteal flap, which enables precise bone harvesting with a rongeur. During the procedure, it is crucial to prevent pterygoid plates, maxillary sinus, molar teeth, and a larger palatine canal to prevent complications (see Figure 2).

The symphysis is the location that produces the most intraoral bone [14]. To gain access to this area, a canine-to-canine vestibular incision must be made at least 3 mm from the mucogingival junction. The periosteum is raised, and the osteotomy is made 10 mm below the incisor tooth’s apex.

**Figure 3:**
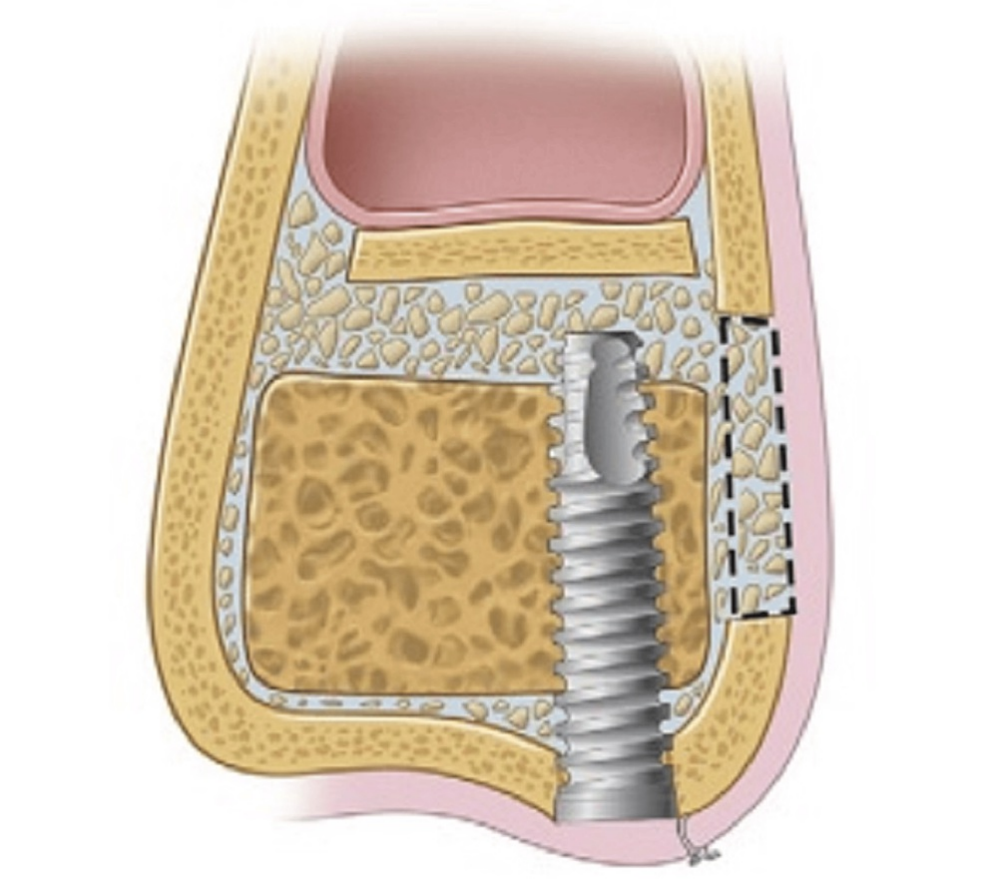
Diagram showing the lateral wall of the maxilla rotated medially into the sinus, which is optional. The bone graft material is placed into the sinus, either in particulate material or block form, to support the implant. Ideally, the block grafts should engage the superior surface of the implant. Source: Stern and Green (2012) [2]; reproduced with permission from Elsevier

A trephine with a collecting instrument positioned along the suction line is an appropriate instrument for bone harvesting. The cortical plate may be eliminated if required, and the bone marrow can be extracted (Figure 3). During the procedure, the mental nerve must be avoided, and the patient should be made aware of the enhanced hazard of V3 paresthesia brought on by this method. Related to this, bone can be extracted from the third molar sites in the mandible and posterior maxilla using a scraping tool by collecting a container coupled to the suction.

Tatum first demonstrated the lateral window technique by utilizing a modified Caldwell-Luc method [15]. In this surgical approach, osteotomies are used to create a bone window that is then either removed or turned medially without rupturing the membrane of the sinus. First, a posterior superior alveolar nerve block, a superior anterior alveolar nerve block, and palatal infiltration are used to administer local anesthesia with epinephrine. A local anesthetic may be utilized in conjunction with general anesthesia or intravenous sedation, if necessary. Steroids and antibiotics are often given as preventative measures prior to the surgery, although the decision to use perioperative steroids and antibiotics should be at the discretion of the surgeon. Before beginning the procedure, it is uncertain whether preoperative administration of medication is beneficial or not. Therefore, the surgeon should carefully weigh the potential risks and benefits before administering such drugs. Before performing the incision, the patient is instructed to rinse his mouth with a 0.12% chlorhexidine solution.

**Figure 4:**
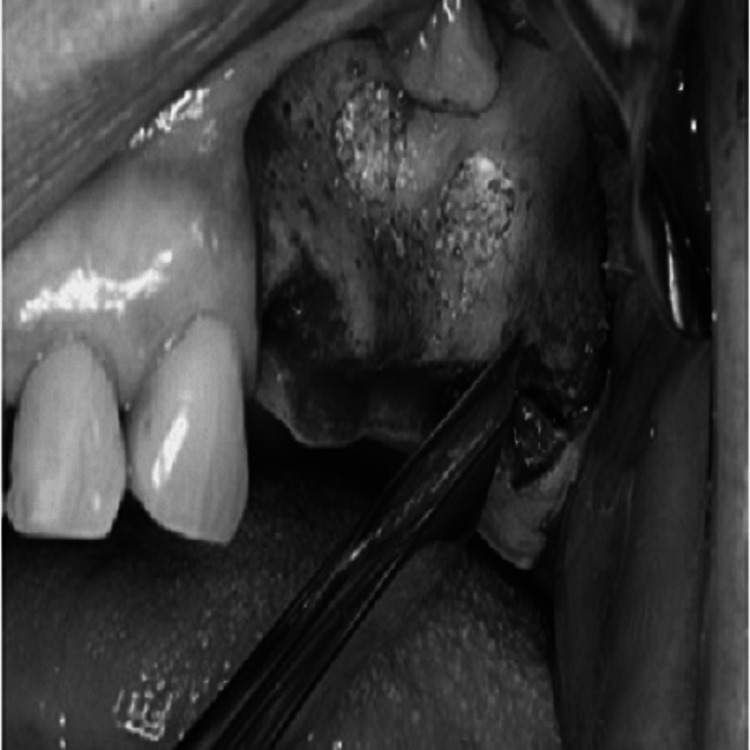
Bone harvesting Source: Stern and Green (2012) [2]; reproduced with permission from Elsevier

**Figure 5:**
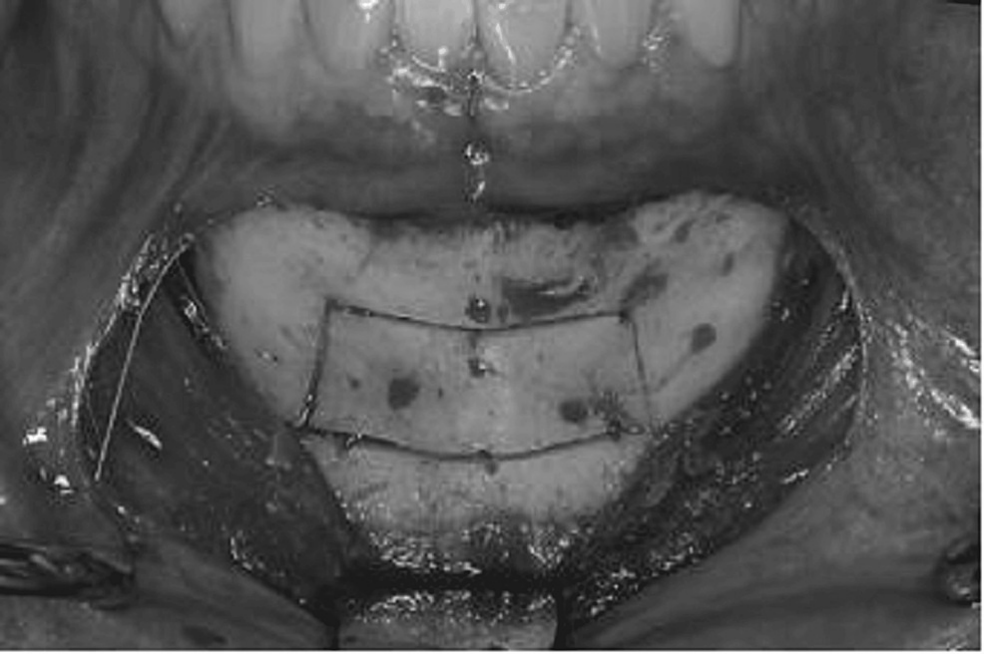
The unicortical osteotomies form a rectangular outline in the symphysis Source: Stern and Green (2012) [2]; reproduced with permission from Elsevier

From maxillary tuberosity to the position immediately anterior to the anterior edge of the sinus, the crestal incision is created. In order to expose the sinus without impairing the sinus window, vertical releasing incisions should also be performed on the anterior and posterior sides to the depth of the vestibule. The maxilla lateral wall should then be revealed by elevating a full-thickness mucoperiosteal flap (Figure 4). Next, four linear osteotomies should be performed using a #6 or #8 round bur, starting with the inferior horizontal osteotomy, which must be performed as near to the sinus floor as feasible, a maximum of 2-3 mm above the floor, and extending posteriorly from the region of the first or second tooth to the anterior edge of the maxillary sinus (Figure 5).

**Figure 6:**
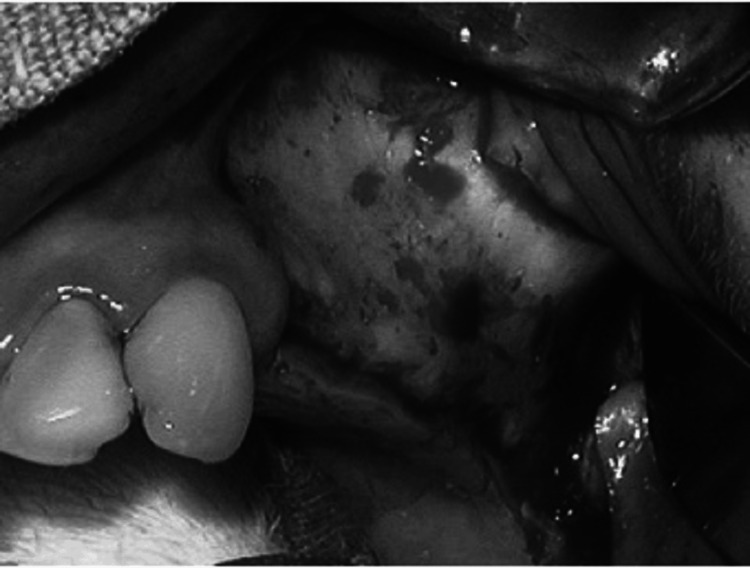
Incision and mucoperiosteal flap reflection Source: Stern and Green (2012) [2]; reproduced with permission from Elsevier

During the procedure of performing osteotomies, it is crucial to use a gentle touch and a brushing stroke to prevent any damage to the Schneiderian membrane. When carrying out the procedure in the presence of bicuspid teeth, extra care should be taken to avoid causing any harm to them, and the osteotomy must be limited to 4 mm from the distal aspect of the tooth. The next step involves performing the superior horizontal osteotomy at a height where augmentation is planned. Finally, the posterior and anterior vertical osteotomies are formed to connect the inferior and superior osteotomies. The osteotomies must run parallel to the lateral wall of the nose and the anterior edge of the maxillary buttress (or maxillary tuberosity), respectively (Figure 6).

**Figure 7:**
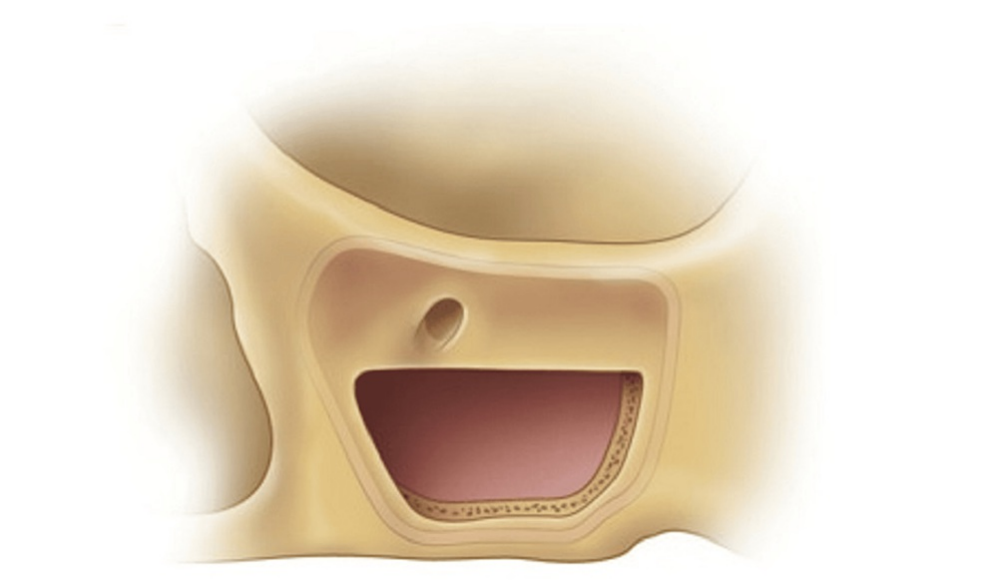
Diagram depicting the ideal location of the sinus window preparation of the lateral maxillary wall. The inferior ostectomy should be approximately 1 mm above or level with the floor of the sinus. The posterior ostectomy should be at the corner of the maxillary buttress. The anterior ostectomy should be adjacent to and parallel to the lateral wall of the nose, and the superior ostectomy should be at the height of the intended graft. Source: Stern and Green (2012) [2]; reproduced with permission from Elsevier

When the window is formed and the membrane is visible, any attached bone is taken out or rotated medially. If the bony window is rotated inwardly, it develops the floor of the maxillary sinus. To elevate the Schneiderian membrane, the process should begin by lifting the margins with caution and then progressively increasing the elevation. Over-elevation of one area should be avoided, as it can lead to perforation. The use of broad-based curettes or freers is recommended for membrane elevation. If possible, the membrane should be raised greater than the superior osteotomy. It is essential to lift the Schneiderian membrane above the superior osteotomy to avoid undue strain on the bone graft substance (Figure 7).

**Figure 8:**
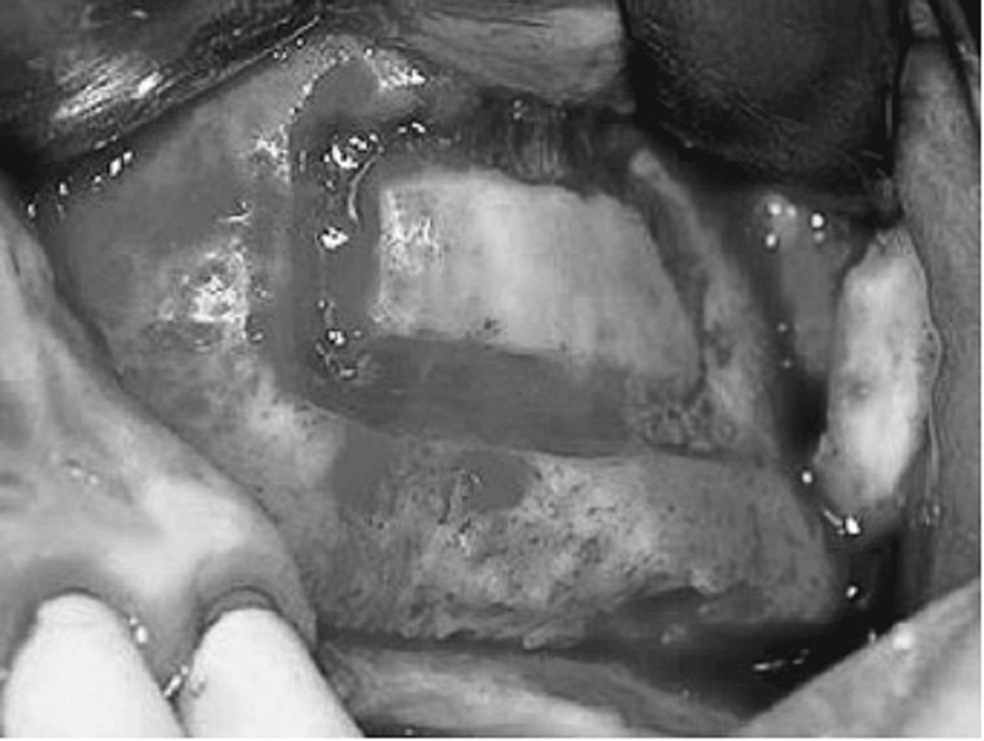
Complete quadrilateral osteotomy Source: Stern and Green (2012) [2]; reproduced with permission from Elsevier

**Figure 9:**
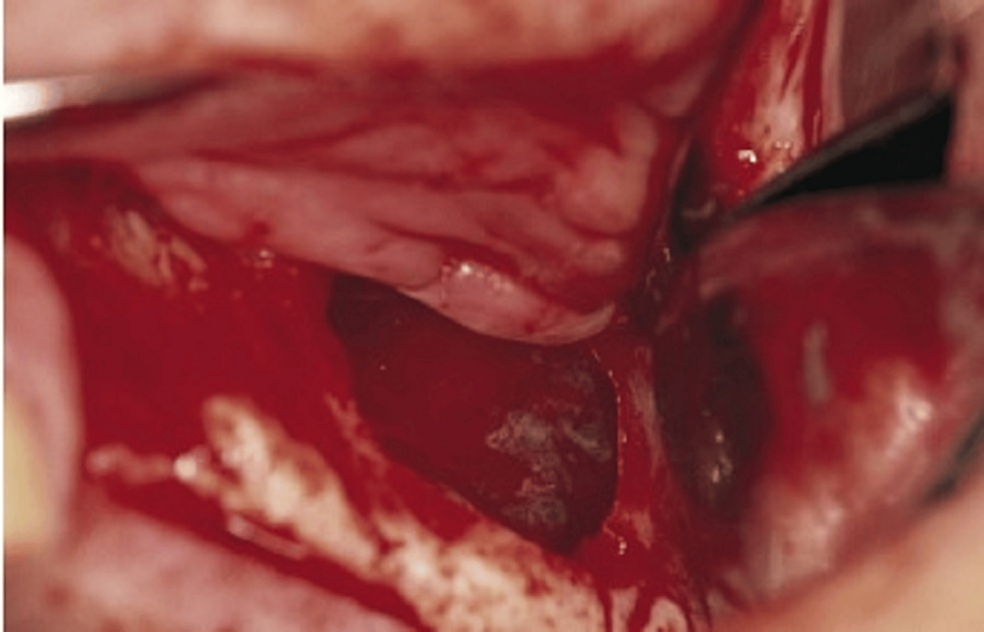
The membrane is carefully elevated and reflected medially into the sinus. Source: Stern and Green (2012) [2]; reproduced with permission from Elsevier

**Figure 10:**
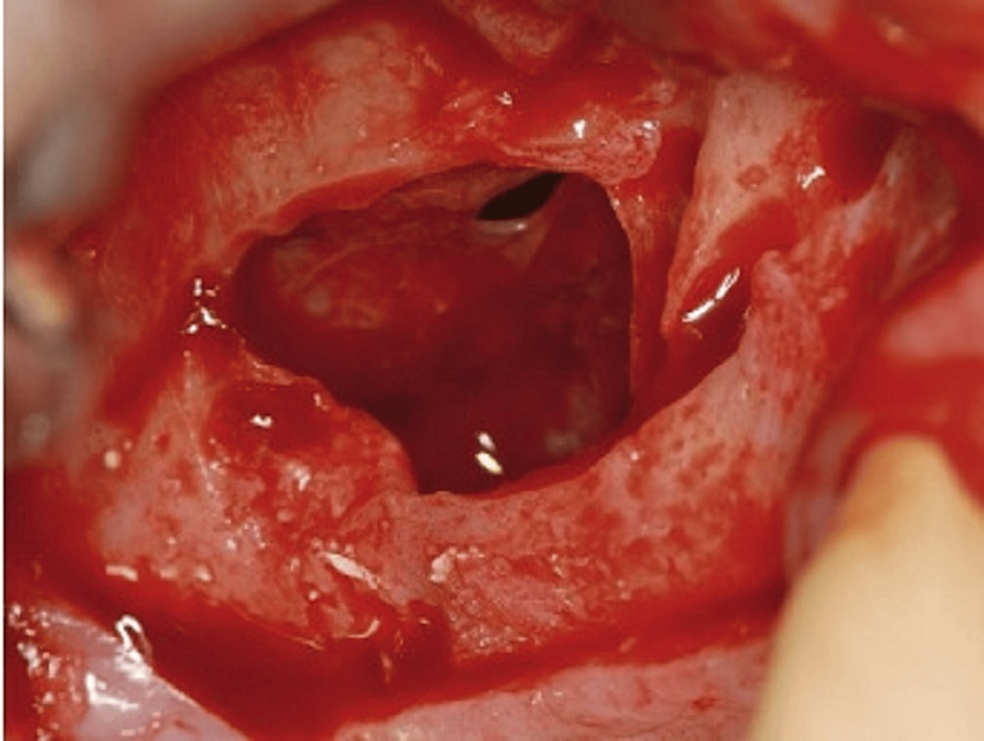
The crestal incision is combined with anterior and posterior vertical release incisions to allow for exposure of the lateral wall of the maxilla. The lateral wall of the sinus is rotated medially with membrane reflection. A small perforation is seen. Source: Stern and Green (2012) [2]; reproduced with permission from Elsevier

Sinus membrane perforation is possible during the procedure, which can lead to complications (Figure 8). Small perforations may not require treatment, but large ones should be patched with a collagen membrane, or the procedure should be aborted. If the treatment is unsuccessful, it should not be repeated for at least four to six months. After raising the membrane, bone graft material is positioned anteriorly and inferiorly beneath it, making sure not to overpack it (Figure 9). To account for volume loss, an extra 20% of bone graft material must be injected. After that, the mucoperiosteal flap is moved and sutured. Six months following the sinus lift treatment, implants can be inserted if there is enough alveolar bone to support them, and bone graft substance can be packed around them (Figure 10). Patients should be prescribed postoperative antibiotics and decongestants for two weeks and advised to take sinus precautions such as not blowing their nose and coughing or sneezing with their open mouth.

**Figure 11:**
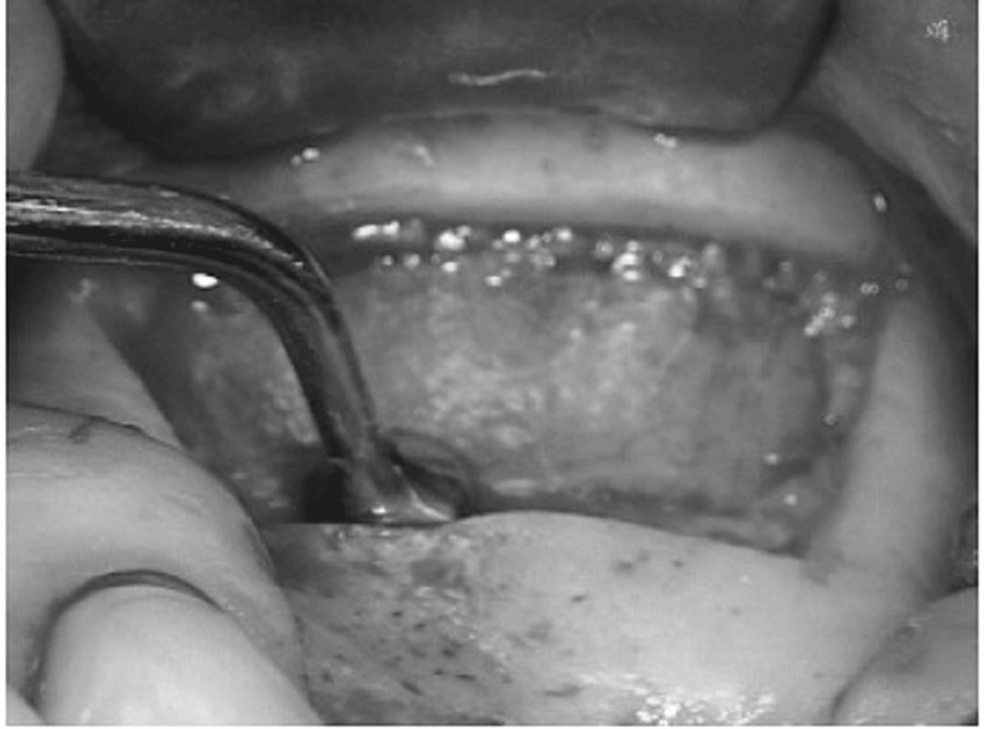
Incision and mucoperiosteal flap reflection Source: Stern and Green (2012) [2]; reproduced with permission from Elsevier

Piezoelectric technology is an ultrasonic tool utilized for making osteotomies. This scheme has a higher power than traditional ultrasonic instruments, enabling the creation of osteotomies in thicker, more compact cortical bone without cutting soft tissue. The benefit of this system is to decrease the hazard of perforating the sinus membrane. The piezoelectric tool may also help in the rise of the sinus membrane, especially in robust areas of thin membranes and bone. This system has various inserts, from osteotomes to diamond-cutting inserts to those that aid in lifting the sinus membrane. To elevate the membrane, the endosteum is separated from the bone, and the piezoelectric cavitation is subjected to the hydropneumatic pressure of a physiological saline solution (Figure 11) [16].

A research study conducted by Vercellotti et al. [17] involved creating 21 bony window osteotomies in 15 patients using the Mectron Piezosurgery System with inserts that had a vibration from 60 to 210 mm and power exceeding 5W. Every osteotomy was carried out while being irrigated by a surgical system pump. After the flap was reflected, the bony window was formed by a piezoelectric scalpel, and then the membrane elevator tip was utilized, beginning at the apex and moving to the distal and mesial aspects. Attention was focused on the sinus floor, where the membrane was raised to limit the harm of perforation. In this study, all sinus augmentations utilized autogenous bone grafts and platelet-rich plasma. The research found just one membrane perforation in 21 patients, yielding a success rate of 95%.

**Figure 12:**
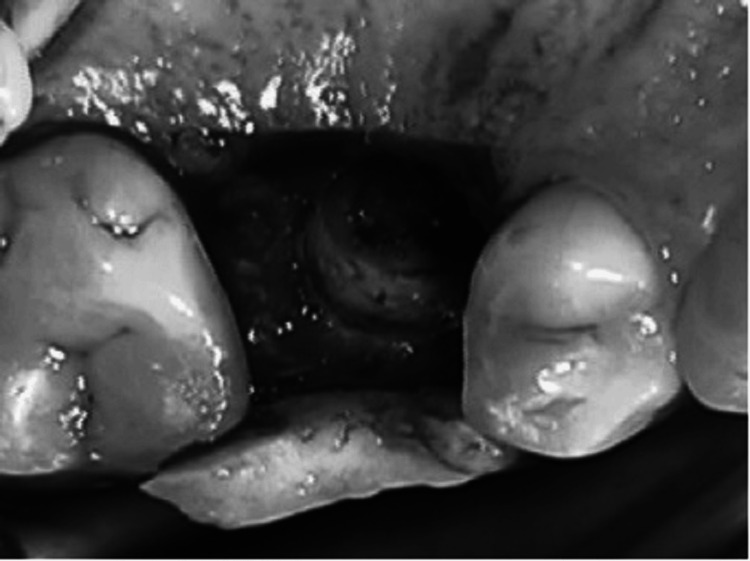
Trephined bone core partially intruded into the sinus cavity Source: Stern and Green (2012) [2]; reproduced with permission from Elsevier

The sinus intrusion osteotomy is recommended for cases where there is a minimum of 5-6 mm of alveolar bone available. This method was demonstrated to increase bone height by 4-8 mm, but it is best suited for situations where having enough bone for implant stabilization and minimal bone height are required [18]. In 1994, Summers [19] originally reported the method, which entails creating a crestal incision, bone prepping, and raising the sinus by a few mm. During the procedure, not only is bone compacted apically and the sinus elevated, but bone is also compacted laterally with progressively larger osteotomes (Figure 12).

**Figure 13:**
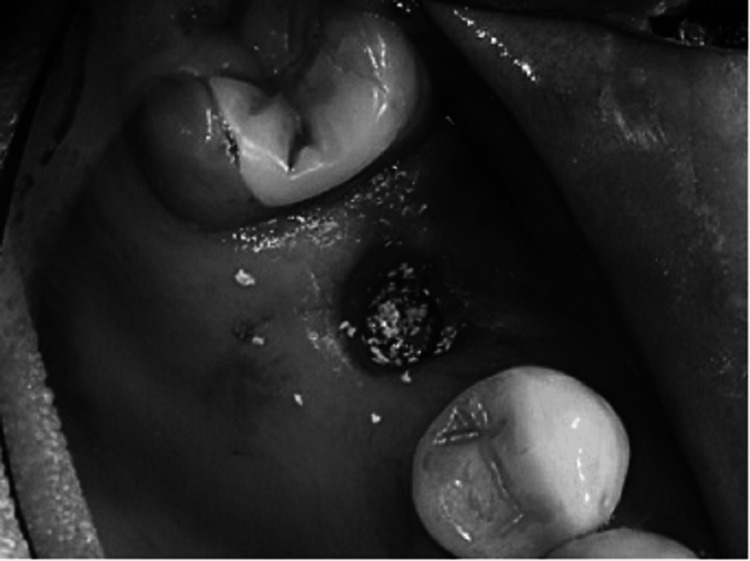
Graft placed through the implant receptor site into the sinus cavity Source: Stern and Green (2012) [2]; reproduced with permission from Elsevier

During the procedure described by Summers, a crestal incision is made, and implant drills are utilized to construct an osteotomy, leaving 1 mm of bone within the sinus membrane and site. Then, sequential osteotomes of progressively increasing diameter are utilized to determine the depth of the desired implant length, compacting bone apically and laterally, and elevating the sinus membrane. Once the required diameter and length are achieved, the bone graft substance is put in the prepared site’s apical portion (Figure 13). The implant is located at the desired length, ensuring its stability. The main closure is then completed by adding a cover crew. The healing abutment can be connected to the implant, and it can be exposed after four to six months of healing. Similarly, Komarnyckyj and London [20] also observed a 95.3% success rate when performing this process on 16 patients and placing 43 implants. This study demonstrated a mean bone increase of 3.25 mm throughout the follow-up period of nine to 47 months.

**Figure 14:**
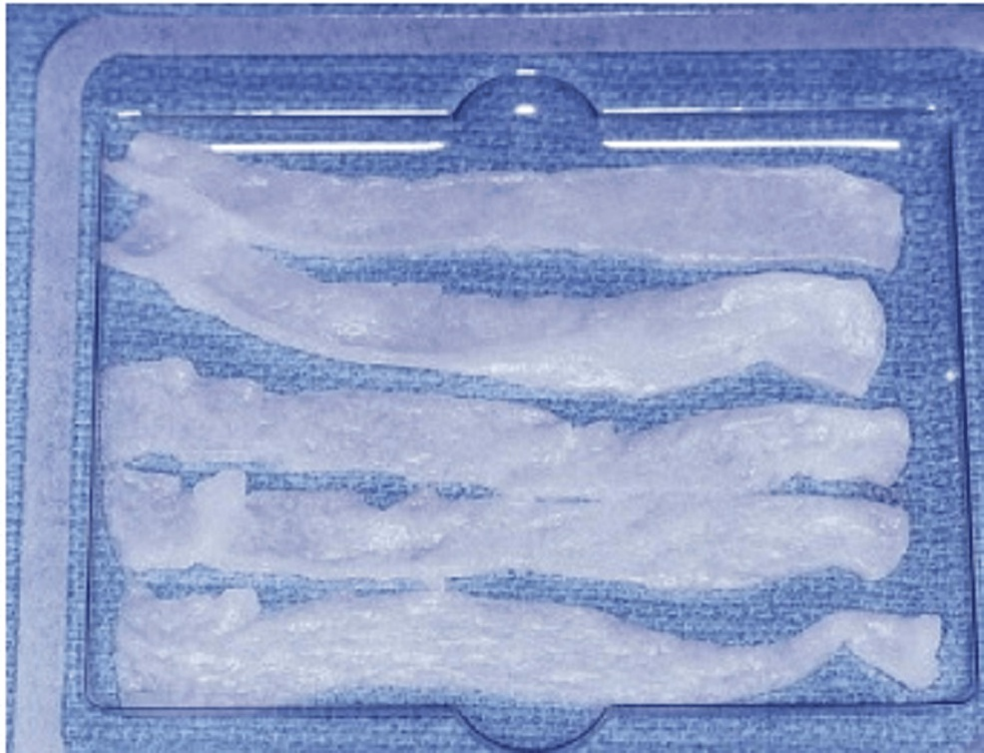
BMP is placed on a collagen sponge, and the sponge is cut into five or six strips. Source: Stern and Green (2012) [2]; reproduced with permission from Elsevier

**Figure 15:**
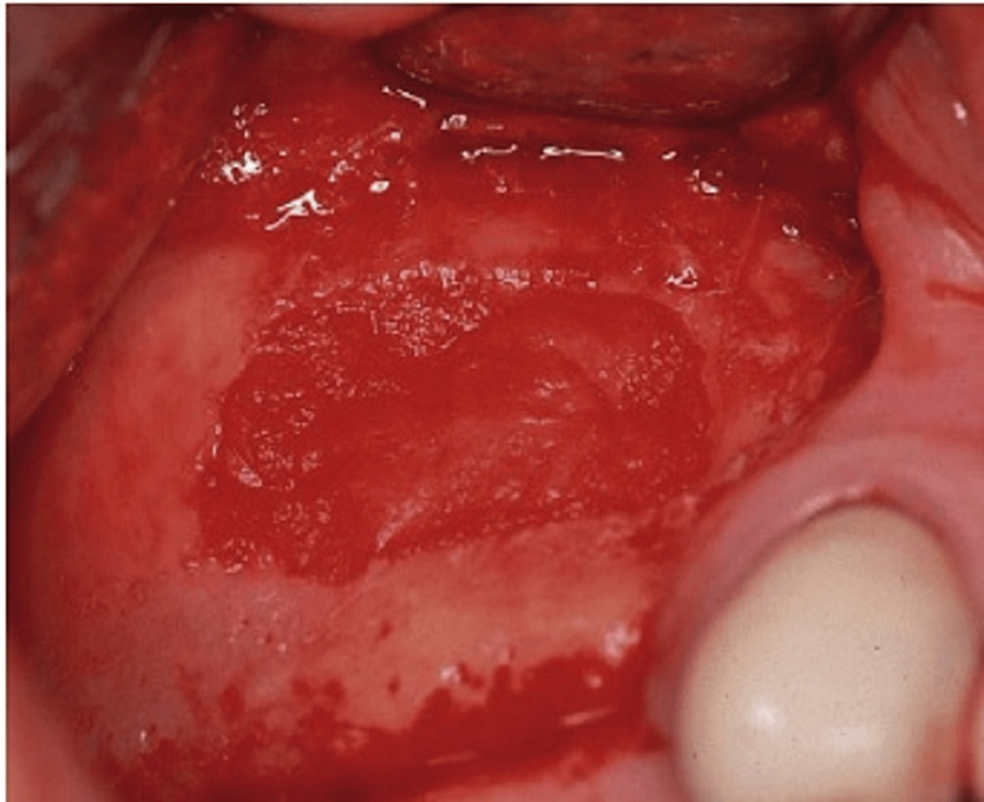
A BMP-impregnated collagen membrane is placed into the sinus with no membranes used to cover the sinus graft site. Source: Stern and Green (2012) [2]; reproduced with permission from Elsevier

BMP is an option for bone graft material for enhancing the maxillary sinus, which is gaining popularity in the field. BMPs are altering growth factors that have bone-inductive characteristics, and two recombinant human proteins, rhBMP-7 and rhBMP-2, are currently available [21]. BMPs offer several advantages over bone graft materials, including no morbidity at the harvest site, ease of use, increased soft tissue healing, and the ability to be used in people who are not candidates for autogenous grafts [10]. BMPs come in powder form and can be mixed with sterile water and applied to the carrier during surgery. Collagen is the most commonly used carrier material for maxillary sinus augmentation, but it does not have mechanical strength and should be utilized in an area with borders in every dimension [10]. The preferred method for utilizing BMP-2 for maxillary sinus augmentation is the lateral window approach, although there is limited evidence for its success with the sinus intrusion osteotomy technique. The procedure involves administering local anesthesia, making an incision, raising a full-thickness mucoperiosteal flap, creating bony osteotomies, and elevating the sinus membrane as earlier described. If a perforation in the membrane occurs, it is not mandatory to repair it when using BMP-2, but the surgeon may choose to do so. The BMP is provided in lyophilized powder form and reconstituted using sterile water, following the manufacturer’s instructions. The reconstituted BMP is loaded into a sterile syringe and applied evenly to the collagen sponge (Figure 14). It takes at least 15 minutes to let the liquid settle so that the BMP adheres to the sponge before it is cut into 15-mm strips and located among the bony floor and membrane of the sinus (Figure 15). Chromic gut sutures are used to achieve primary closure.

A one-week course of antibiotics is prescribed, and the patient is advised to take sinus precautions. The patient must be notified about the likelihood of significant swelling. A postoperative panoramic radiograph may be taken after four months to assess bone formation, and implants may be located six months prior to the process. Boyne et al. [22] conducted an early study that demonstrated the successful usage of rhBMP-2 in 12 patients who received the material in their maxillary sinus. The average bone height was 8.51 mm. The most common postoperative adverse impacts were facial swelling, pain, redness, and rhinitis.

Implants were inserted in the increased sinuses 12 weeks later and permitted to integrate for three months, in accordance with research comparing rhBMP-2 to anterior iliac crest grafts in 30 rabbits [23]. The rhBMP-2 group saw the largest mean vertical bone growth, and both groups’ bones had comparable quality, according to the research. It is essential to highlight, however, that recombinant BMP is contraindicated in individuals with hypersensitivity to proteins, carriers, or any other formulation substances. In addition, it should not be applied to people who have active cancers or are receiving cancer treatment, to those who have tumors that have already grown or been removed, to skeletally immature people, to women who are pregnant, or to people who have active infections. Patients who do not demand a separate way to get bone graft material may find that BMP is a good alternative.

Postoperative instructions and management

After surgery, it is important to provide patients with both printed and oral instructions regarding postoperative care. The patient should be advised to avoid consuming rough or hard foods, which can damage sutures and cause wound dehiscence. Additionally, sinus precautions should be taken, which include avoiding any activity that can result in sudden pressure variations in the sinus, like sneezing and nose blowing. If the patient needs to sneeze, they should do so with an open mouth to direct pressure away from the sinus. It is important to inform the patient about common postoperative symptoms, such as soreness, which is normal as well as expected for numerous days post-surgery. According to the postoperative instructions, it is common for a few patients to go through bleeding from the surgical incision for up to 24 hours after the sinus lift surgery. However, this bleeding may seem worse than it is because it can mix with saliva. The instructions advise that the patient should swallow the blood rather than expectorate it. If the bleeding becomes bothersome, it can be controlled by direct wet gauze pressure. If the bleeding persists after two uses of gauze for every hour or if the volume is concerning, the patient must notify the doctor. In addition, occasional skin bruising and swelling are normal after a sinus lift surgery.

Complications and their management

Schneiderian membrane perforation is the most frequent complication of maxillary sinus lift surgery (Table 2). In prospective observational, uncontrolled research, 70 patients had a total of 212 implant loadings after undergoing 81 sinus lifts. A total of 44% of sinuses have been intraoperatively perforated, but they were healed, and the operation was finished without difficulties. Moreover, 2% of sinuses experienced perforations so severe that the treatment had to be terminated. Furthermore, 33% of perforations were found in sinuses where septae were visible on preoperative radiographs, and 52% of the sinuses with septae were perforated. Two of the 36 holes were so serious that the surgery had to be terminated.

**Table 2:** Common complications of sinus lift surgery Source: Stern and Green (2012) [2]; reproduced with permission from Elsevier

Complication	Treatment
Graft exposure	Gentle daily normal saline irrigation allows for creeping epithelialization
No graft present after the maturation phase	Assess for a possible etiology and retreat
Paresthesia CN V2 distribution immediately postop	Medrol dose pack if no contraindications
Facial swelling two to three days post-surgery	No treatment; normal postop
Severe facial ecchymosis appearing one to three days postop	No treatment; normal postop
Facial pain and swelling one-week postop	Clinical examination; CT scan; consider antibiotics
Swelling and acute onset	Possible air-emphysema; antibiotics; reinforce nasal precautions

Common treatments for sinus perforation involve doing nothing if the perforation is <2 mm in diameter and placing a slowly resorbing collagen membrane if the perforation is >2 mm in diameter. In one patient who presented with acute sinusitis following implant placement, postoperative complications involved the extrusion of a graft into the sinus cavity. Following medical as well as surgical remedies, the infection cleared up, and implants were restored. Persistent peri-implantitis and a peri-implant cyst were among the issues that arose later. Importantly, even though membrane perforations have been linked to postoperative complications like local infection, pain, and swelling, there is no correlation between intraoperative perforations and long-term implant survival. This research revealed an overall survival rate for seven years of 95.5% for implants implanted in grafted sinuses. Noteworthy is the fact that five of the nine unsuccessful implants were implanted in heavy smokers. Instances of chronic infections resulting in severe sinusitis and the potential for graft extrusion, exposure, and/or failure are uncommon. Treatment usually depends on the symptoms that are currently present and may include antibiotics, drainage of surgical debridement, or a Caldwell-Luc technique [24-26].

## Conclusions

The maxillary sinus lift is a widely accepted standard treatment for edentulous maxillas, having been in use for the last 30 years. A very frequent occurrence is pneumatization of the maxillary sinus as a result of posterior maxillary tooth loss. Significant maxillary atrophy prohibits implant insertion in this location. Sinus augmentation was utilized for decades to prepare these areas for dental implant insertion.

Transalveolar and lateral antrostomy procedures are the two most common methods for enhancing posterior maxillary vertical bone height. The clinical and radiographic evaluations identify the most appropriate treatment strategy for every clinical condition. Both methods were demonstrated to have greater success rates. There are few absolute contraindications to the sinus lift procedure, with most being relative contraindications. However, practitioners must be well versed in how to address them. Adequate preparation, knowledge, and experience make maxillary sinus augmentation and elevation a beneficial process for patients, with an expected outcome.
